# Effect of the Frustration of Psychological Needs on Addictive Behaviors in Mobile Videogamers—The Mediating Role of Use Expectancies and Time Spent Gaming

**DOI:** 10.3390/ijerph17176429

**Published:** 2020-09-03

**Authors:** Andrés Chamarro, Ursula Oberst, Ramón Cladellas, Héctor Fuster

**Affiliations:** 1 Department of Basic, Developmental and Educational Psychology, Autonomous University of Barcelona, 08193 Bellaterra, Spain; ramon.cladellas@uab.cat; 2 Serra Hunter Programme, Generalitat de Catalunya, 08003 Barcelona, Spain; 3 Department of Psychology, Blanquerna School of Psychology, Education and Sport Sciences, Ramon Llull University, 08022 Barcelona, Spain; ursulao@blanquerna.url.edu; 4 School of New Interactive Technologies, University of Barcelona, 08007 Barcelona, Spain; hectorfuster@gmail.com

**Keywords:** smartphone, videogames, mobile gaming, casual videogames, addiction, self-determination theory, need frustration

## Abstract

Casual videogames (CVGs), played on smartphones, are becoming increasingly popular, especially among females and adults. Whereas the addictive potential of online (computer) videogames is well-established, there is yet insufficient evidence for Internet gaming disorder (IGD) in mobile gamers and for the mediating role of some mechanisms involved. The aim of this study is to investigate the impact of the frustration of psychological needs on mobile videogamers’ addictive behavior as well as the role of cognitions (game-use expectancies) and behaviors (time spent playing) through a hypothesized serial mediation model, while controlling for important correlates, such as game genre, age, gender and payment during play. A total of 471 mobile game users (211 males) with an average age of 21.73 replied to an online survey containing sociodemographic and game variables, the Need Satisfaction and Frustration Scale (NSFS), the Internet Gaming Disorder Scale-Short Form (IGDS9-SF) and a slightly modified version of the Internet Use Expectancies Scale (IUES). The results corroborate the negative effects of need frustration on IGD among mobile gamers and clarify the role of time spent playing and game-use expectancies in the development of IGD, highlighting the important role of cognitions in this relationship. We conclude that both the time spent playing and game-use expectancies should be important targets for clinical interventions, even though they are not included in the diagnostic criteria.

## 1. Introduction

The use of smartphones is constantly increasing all over the world. In Spain, according to the Spanish Institute of Statistics [1], Internet access is present in 91.4% of households and mobile phones are found in 73.7%; in 24.7%, mobile phones are the only communication device. Smartphones are particularly attractive to young people, who start using them earlier and earlier. By age 10, 22.3% of children have access to or possess a smartphone, and by age 15, 93.8% do. Smartphones are frequently used for leisure. Twenty-seven percent of European smartphone users play videogames on their smartphone, which is the same as the percentage of Europeans who play videogames on their computers or consoles [2]. In the United States, 60% of adults play videogames on their smartphone [3]. These figures seem to be increasing, facilitated by access to high-speed Internet and mobile Wi-Fi networks and the popularity of the devices and apps [4]. Mobile gaming is very entertaining [5] and convenient; users can play almost anywhere and at any time against other players online, enabling them to create or maintain social relationships [6].

The possibility to access videogames on a smartphone means that gaming does not depend on the user having access to a computer or console. This platform change has made videogames even more popular and has also led to a change in user profiles. Whereas Internet gaming is a predominantly male domain, 46.7% of mobile gamers in Europe are women [2]. According to the Spanish Association of Videogames [7], the smartphone is the preferred gaming platform for Spanish women and they represent 42% of gamers. Mobile gamers play an average of 6.7 h/week on their smartphones. There is also a shift in game genres; casual videogames (CVGs, videogames that are typically distinguishable by their simple rules and do not require excessive commitment in contrast to more complex games) are becoming the most popular videogames, especially among older videogamers [8]. CVGs are integrated into an online social network that facilitates interaction with others. Players may use these games to pass the time while waiting or when bored [9].

Gaming behaviors are being influenced by the popularity of mobile gaming. However, most of the research about the consequences of intensive gaming has been designed and tested on users who game on personal computers. Recent comparisons on usage patterns and their consequences, in terms of addictive behaviors, are descriptive and have been conducted without a theoretical basis [10]. It is still unclear how psychological factors and usage patterns interact in problematic gaming on mobile devices. This study aims to explore how psychological factors are combined with usage patterns to explain problematic gaming on mobile devices. We expect that our findings will be useful for optimizing treatments for problematic gaming.

### 1.1. Detrimental Effects of Videogaming on Well-Being

Increased time spent and frequency of use can lead to negative consequences. Two of the most discussed consequences in the scientific literature are Internet gaming disorder (IGD) [10,11] and aggression in boys who play certain action games. IGD has recently been introduced in the nosological manuals as a disorder under the heading of addictive behaviors. The Diagnostic and Statistical Manual of Mental Disorders, Fifth Edition [12] places IGD in Section III (disorders requiring further investigation) and the International Classification of Diseases 11th edition (ICD−11) considers IGD among non-substance addictions. IGD is an addictive behavior that does not involve the ingestion of a psychoactive substance and is mainly characterized by recurrent and persistent participation in online video games, leading to clinically significant distress. IGD has been associated with a wide spectrum of psychological problems including depression, anxiety, social phobias, poor school performance and lack of sleep [13,14,15]. Other studies have linked IGD to poor psychological well-being, low life satisfaction, low self-esteem, loneliness, substance abuse and personal problems [16].

One of the risk factors for IGD is the user’s motive for gaming. Risk is higher when the motive is to alleviate stress, and/or to escape from reality. These motives appear to be linked to certain game genres [17]. For instance, players of first-person shooter games (FPS) play to gain competence and control [18] but a third of players of massively multiplayer online role-playing games (MMORPG) play to alleviate stress and to cope with the hassles of daily life [19]. Wanting to escape from reality is an important motive linked to problem use of videogames [20] and MMORPG [21].

However, there are important gaps in the research on the effects of playing videogames, because few studies have taken into account the gaming platform or the game genre [22]. In fact, most research on the health effects of videogames has focused on personal computers [23], specially MMORPGs [24] or multiplayer online battle arena games (MOBAs) [25]. But smartphone CVGs like Candy Crush Saga can also be highly addictive, because they can be played at any time and in any place, for entertainment, connecting with friends, enjoying the feeling of victory, relaxing or “killing time,” and because the use of these games is linked to loneliness and lack of self-control [4]. According to these authors, mobile games will become increasingly preferred by most users. Evidence has shown that on one hand, videogames make it possible to satisfy basic psychological needs [26]; but on the other hand, the negative consequences of videogame use are associated with difficulties in self-regulation. As self-regulation and need satisfaction (and need frustration) are core elements of self-determination theory (SDT) [27], it makes sense to use this theory as a theoretical framework for studying the consequences of mobile gaming.

### 1.2. Self-Determination Theory and Videogames

According to SDT [28] people tend toward psychological growth and the satisfaction of their basic psychological needs. This drive makes it possible for the personality to develop and for people to self-regulate their behavior. These basic psychological needs are autonomy (i.e., feeling of being the origin of one’s own behaviors, acting under one’s own volition), competence (i.e., feeling effective and skilled) and relatedness (i.e., sensing connection to peers, feeling understood and cared for by others). They represent the “psychological nutriments that are essential for ongoing psychological growth, integrity and well-being” [28] (p. 229). According to Ryan and Deci [28], need fulfillment is a driver of human behavior; it is intrinsically motivating, makes activities enjoyable and culminates in positive psychological outcomes. A meta-analysis concluded that need fulfillment leads to improved mental health (e.g., less depression and anxiety and higher quality of life) and more health-conducive behaviors (e.g., tobacco abstinence, more exercise, healthier diet, adaptive sleep patterns) [29]; it is also related to enjoyment of leisure activities such as videogames [30]. In sum, need satisfaction is universally linked to well-being across life domains [31]. In consequence, basic psychological need satisfaction in everyday life is accompanied by experiences of choice, energy and enjoyment.

However, basic psychological needs may also be frustrated, such that individuals experience active impediments to the satisfaction of one or more of the needs in their daily lives [32]. Importantly, there is a difference between a deficit in need satisfaction (i.e., when somebody does not feel sufficiently recognized) and need frustration (i.e., when somebody is excluded or rejected by others) [33]. Need frustration includes feeling forced to act in a way that is uncomfortable or uncharacteristic (autonomy frustration), being made to feel inferior in terms of effectiveness (competence frustration) and sensing rejection or isolation from family and/or peers (relatedness frustration). In consequence, need frustration does not merely represent a lack of satisfaction but a separate construct that exhibits different relationships to external outcomes [27,33]. Frustration of basic psychological needs has only recently been studied as separate from low need satisfaction and has been proposed to be related to the development of psychopathology [34]. Need frustration is especially predictive of poor well-being—disengagement, poor sleep, elevated blood pressure, disordered eating and obsessively pursued behaviors [35].

In sum, need frustration presents a risk factor for a disordered pattern characterized by long hours of compulsive, tense and unenjoyable play [36]. It has been suggested [34] that need frustration may be an underlying factor in IGD because of its contribution to time spent gaming. Interestingly, pathological gaming is most likely to occur when satisfaction of the three basic human needs is low in the gamer’s real world but high in videogames [37]. Thus, need frustration may be a potential risk for this behavioral pattern, which implies a pressure toward mobile gaming and a loss of control over it. Interestingly, need frustration in both face-to-face and online contexts is positively associated with IGD scores [38]. IGD scores are shown to increase with daily need frustration, because it undermines self-control, which in turn contributes to greater IGD [30].

In sum, SDT can be a useful model for examining the relationship between need frustration and negative well-being outcomes for videogamers [39]. To examine this relationship, a series of variables that could act as mediators and/or confounding variables have to be considered and statistically controlled, as explained in the following sections.

### 1.3. Game-Use Expectancies, Game Genre, Age, Sex and Payment

Specific individual predispositions for using Internet applications act as mediators in the onset of the addiction process [40], for instance cognitive biases, such as users’ expectancies that certain applications will gratify their current needs and desires. Unlike motives, which are considered relatively stable and imply a tendency to use technology, expectancies are ideas or thoughts about the outcomes of using specific applications or devices, in terms of experiencing pleasure or avoiding negative feelings [41].

Both positive expectancies (i.e., to experience pleasure) and avoidance expectancies (i.e., to escape from reality) lead the user to experience gratification and reinforce his or her motivation for using the application. As a result, the individual decides to use the Internet more often, leading to a type of reinforcement circle and resulting in an addictive use [40]. In the case of IGD, avoidance expectancies mediate the relationship between, on one hand, maladaptive personality traits such as negative affectivity, detachment and psychoticism and, on the other hand, addictive symptoms [42]. Since use expectancies have been shown to play an important role in problematic Internet use and in IGD, it is worthwhile to investigate if they play a similar role in mobile gaming. This makes sense, because, normally, gamers play to obtain gratification. Because addicted gamers are characterized by boredom avoidance and a preference for playing on their smartphones [4], they spend a lot of time playing in different places.

In terms of game genres, in MMORPGs, the player selects an avatar that represents him or her and with whom he or she identifies (i.e., autonomy), accomplishes objectives through adventures (i.e., competence) and socializes with others (relatedness) [43,44]. In these games, participating in a team and competing are the best way to ensure progress in the game and therefore, despite MMORPGs and MOBAs being the games that are most associated with IGD [21], playing does not necessarily have negative consequences [45]. Regarding CVGs such as Candy Crush, despite evidence of their addictive potential [4], little attention has been paid to the psychological aspects of smartphone gaming [46].

There do not appear to be sex differences in problematic Internet use, given that both sexes are equally subject to use expectancies [40]. However, in the non-pathological use of videogames, boys are more motivated to play than girls [47], especially because of the challenge component that they include [48]. In this line, research suggests that FPS players are mostly male [26,49]. In general, males report spending more time gaming than females [34,50] and show higher levels of addiction [51].

In contrast, females do not generally see videogames as an activity that satisfies their basic needs [34] but prefer videogames that involve socializing [47] and therefore are more likely to use CVGs [6,52]. Thus, being female might be a risk factor for playing mobile games excessively. Furthermore, mobile videogames are preferred by older adults [4]. In short, previous research suggests that sex and age should be taken into account in studying mobile videogame use.

Time spent playing is a key variable in the problematic use of videogames [53,54] and of smartphones [55]. Gamers with addiction spend more time and more money on videogames than gamers without addiction [56]. However, some authors [57] have pointed out that gamers spend long periods of time online with friends and other members of the community and not all develop IGD. For this reason, time spent on a game is not a clear indicator of problematic use [38], given that it can vary widely across different types of videogames [57]. Excessive time is a necessary but not sufficient, condition for addiction that is reflected in environmental variables [55]. Other authors have highlighted that the amount of time played is less relevant for assessing IGD than the negative consequences the activity might produce [58,59,60]. These findings notwithstanding, time spent playing must be taken into consideration, given that it keeps gamers from carrying out other activities [58,61]. Finally, another difference between smartphone gaming and videogaming is the more frequent possibility of payment during the game on smartphone (pay-to-play, pay-to-win). The pay-to-win modality is more frequent in CVGs and represents a way to achieve a clear advantage over the other users who do not pay. It has been argued that, in these models, the boundaries between gaming and gambling tend to disappear, because some young adults spend large amounts of money on these games [58].

### 1.4. Purpose of the Present Study

It appears that gamers can satisfy basic psychological needs by playing videogames [44]. However, some highly-engaged players may experience active impediments to the satisfaction of one or more of their needs, if their gaming keeps them from participating in offline activities, for example, if they cannot carry out organizational activities, exercise leadership, share their experience and communicate both inside and outside the game [26]. In general, there is evidence of a relationship between the frustration of psychological needs and IGD [37]. It is important to examine this relationship in more detail by considering the gaming platform; in this case we analyze mobile gaming. We also consider variables that can mediate the relationship between psychological needs and IGD for mobile gaming—game expectancies and time spent playing. According to Brand et al. [41] a model that describes mechanisms involved in the development and maintenance of addictive behavior should include a person’s core characteristics leading to specific cognitive responses. Thus, when people’s needs are frustrated, they seek ways to satisfy them. This raises expectancies related to specific activities, such as videogaming, either by expecting gratification and joy or by avoiding the negative thoughts associated with the frustrated need. This approach is in line with that proposed by Griffiths and Nuyens [62] who suggest that in order to explain pathological videogame use, we must adopt levels of analysis that consider individual components and also structural traits of the videogames.

**Figure 1:**
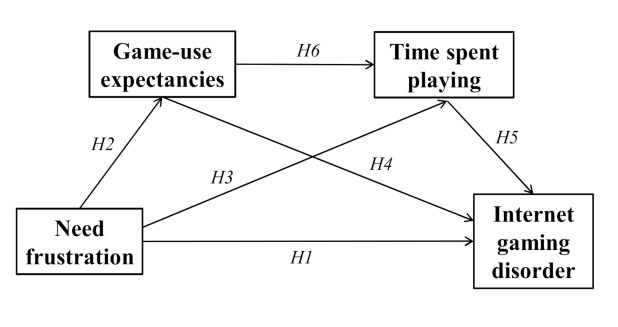
The mediation model.

We aimed to investigate the mechanism and impact of the frustration of psychological needs on mobile videodeogamers’ addictive behavior through a hypothesized serial mediation model. The model hypothesizes a direct effect of frustration of psychological needs on IGD (H1), game expectancies (H2) and time spent gaming (H3). It is also expected that game expectancies (H4) and time spent gaming (H5) will have an effect on IGD. Finally, the indirect effect of need frustration on IGD will be serially mediated by gaming expectancies and time spent gaming, controlling for game genre, age, sex and payment, thus implying an effect of game expectancies on time spent gaming (H6). The mediation model is depicted in Figure 1.

## 2. Materials and Methods

### 2.1. Participants

The convenience sample was composed of 471 participants (260 females and 211 males) answering an online survey presented through Google Forms and distributed by means of online social networking, such as Facebook and WhatsApp. The inclusion criterion was playing mobile videogames at least once a day; respondents not fulfilling this criterion were not allowed to complete the survey. The average age of participants was 21.73 years (SD = 10.10) with a range of 12 to 63 years; there was no significant gender difference with respect to age. All participants gave their informed consent prior to participating by pressing the corresponding button in the online form.

The study is in compliance with the APA ethical principles and the Declaration of Helsinki. Ethical clearance had been obtained from the second author’s university ethics committee.

### 2.2. Instruments

The online questionnaire included:

#### 2.2.1. Sociodemographic and Game Variables

Participants reported their sex, age and aspects related to their use of mobiles games. For preferred videogame genre, participants had to select from the following—(i) casual/puzzle (e.g., Candy Crush Saga or Angry Birds), (ii) strategy/simulation (e.g., Clash of Clans, Lords Mobile, Dragon Mania, Farming Simulator and The Sims), (iii) collectible cards (e.g., Clash Royale and Hearthstone), (iv) gacha games (e.g., Dragon Ball and Dokan Battle), (v) action and adventure (e.g., Battlelands Royale, Mystery), (vi) social casino (e.g., Zynga and Playfish), (vii) sports (e.g., FIFA and True skate) and (viii) role playing (e.g., Neo monsters). The list of genres included examples. This list of genres was based on the 14-genre classification used by Reference [63]. Different game genres are considered to reflect the preferences of different types of players [64]. Participants also reported on their number of gaming sessions per day and the time per session. Game time is a game characteristic associated with IGD [23]. For the analyses, we multiplied the number of daily sessions by the time spent per session in order to calculate the daily time spent gaming. As suggested by Rho et al. [56], participants also reported whether they tended to make in-game purchases and, if so, how much they spent. Spending money during videogames is common, both in free games (i.e., freemium) and paid games (i.e., pay-to-play and pay-to-win). As stated before, players can purchase access to advanced services, achievements and improved experiences, increasing the additive potential of these games [62].

#### 2.2.2. The Satisfaction and Frustration of Psychological Needs

We used the Spanish version [27] of the Need Satisfaction and Frustration Scale (NSFS) [65]. It includes 18 items that measure the satisfaction or frustration of each basic psychological need (i.e., autonomy, competence and relatedness). The introduction was modified to relate specifically to mobile gaming. For each of the three needs, three items measure satisfaction (e.g., I feel that I care about the people around me) and three items measure frustration (e.g., I feel that sometimes others exclude me). Each item is answered using a Likert scale ranging from 1 (completely disagree) to 7 (completely agree). For this study, we used only the frustration subscale, obtaining a single indicator resulting from the summation of the nine items. The reliability (Cronbach’s alpha) of this scale in our study was 0.79.

#### 2.2.3. Internet Gaming Disorder

We measured IGD using the Spanish version [66] of the Internet Gaming Disorder Scale-Short Form (IGDS9-SF) [12]. The IGDS9-SF is based on the nine criteria suggested by the DSM−5 for IGD, which include—(i) preoccupation with gaming; (ii) withdrawal symptoms; (iii) tolerance; (iv) unsuccessful attempts to reduce or quit gaming; (v) loss of interest in previous activities or entertainment as a result of (and with the exception of) gaming; (vi) continuing to game despite knowing the associated psychosocial problems; (vii) deceiving family members, therapists or others about the amount of time spent on gaming; (viii) playing video games to evade or relieve negative moods; and (ix) jeopardizing or losing a meaningful relationship, a job or an educational or employment opportunity due to gaming. The IGDS9-SF assesses the severity of IGD and its detrimental effects, which occur over a 12-month period. Items (e.g., Do you feel the need to spend more and more time playing to achieve satisfaction or pleasure?) are rated on a Likert scale ranging between 1 (never) and 5 (very often). Scores range from 9 to 45; higher scores typically indicate a higher degree of IGD, greater severity of symptoms and greater incidence of problems related to gaming behaviors. The reliability (Cronbach’s alpha) of this scale in our study was 0.88.

#### 2.2.4. Game-Use Expectancies

We used the version adapted for Spanish [40] of the Internet Use Expectancies Scale (IUES) [41]. The scale was originally developed to assess user expectancies related to the Internet, so we modified the introduction to address expectancies related to mobile gaming. The scale consists of eight items assigned to two factors—positive reinforcement (four items; for example, I use mobile video games to experience pleasure) and avoidance expectancies (four items; for example, I use mobile video games to distract myself from problems); the items are answered in a Likert scale ranging from 1 (completely disagree) to 7 (completely disagree). The reliability (Cronbach’s alpha) of this scale in our study was 0.83.

### 2.3. Procedure

We surveyed participants from April to September 2019 using an online questionnaire. Three graduate students posted the link to the questionnaire in as many social media sites as possible (their own sites and those of their acquaintances) asking for collaboration in a scientific study on mobile games and then using snowball sampling. Snowball sampling is a way of finding a large number of potential respondents by finding a few, for example, on social media and online networking sites and then asking them to find others; it is frequently used in online studies.

After responding to the survey, participants were asked to recruit future subjects from among their acquaintances. Participants gave their informed consent by pressing a Yes/No button prior to responding. All study procedures were performed in accordance with the guidelines of the Declaration of Helsinki. The Ethical Committee of Ramon Llull University approved the study protocol (CER URL 2015_2016_007).

### 2.4. Data Analysis

We conducted descriptive analyses and Pearson correlations with SPSS 26.0. The statistical significance of the mediation effects of the serial multiple mediation model tested in the study was investigated by using the ordinary least squares regression method. Analyses were conducted through SPSS macro PROCESS 3.4.1 (model 6) [67], which allowed us to estimate simultaneously the indirect effects of successive mediators in a single model. We generated 5000 bootstrapped samples to estimate the confidence interval of the model effect. A 95% confidence interval without zero indicates statistical significance.

## 3. Results

### 3.1. Descriptive Results

The average frequency of play was 2.42 times per day (SD = 2.69; range 1−19), with an average duration of 24.94 min (SD = 30.06; range 1−259). There were no gender differences with respect to time spent playing. In-game accessories were purchased by 34.5%, with an average of 14.64 euros spent (SD = 21.39), men spending significantly more money (t = 3.460, df = 165.05, *p* = 0.001). In terms of genre, 43.0% of participants preferred CVGs, 23.4% strategy/simulation, 13.4% collectible card, 7.6% action and adventure, 6.8% sports, 3.8% role playing, 2.4% gacha and 1.3% social casino.

As can be seen in Table 1, frustration of psychological needs was positively correlated with game expectancies and IGD. Time spent was positively correlated with IGD and game expectancies was correlated with time spent and IGD. Age was negatively correlated with need frustration, game-use expectancies and IGD; and payment was correlated with all variables except age.

### 3.2. The Mediation Model

To test for serial mediation, IGD was entered as the outcome variable and need frustration as the predictor variable. Age, gender, game genre and payment were entered as covariates and game expectancies and time spent gaming were entered as serial mediators. Regression coefficients for study variables and covariates over mediators and outcome are depicted in Table 2. Serial mediation assumes a causal chain linking the mediators, with a specified direction of causal flow [67].

**Figure 2:**
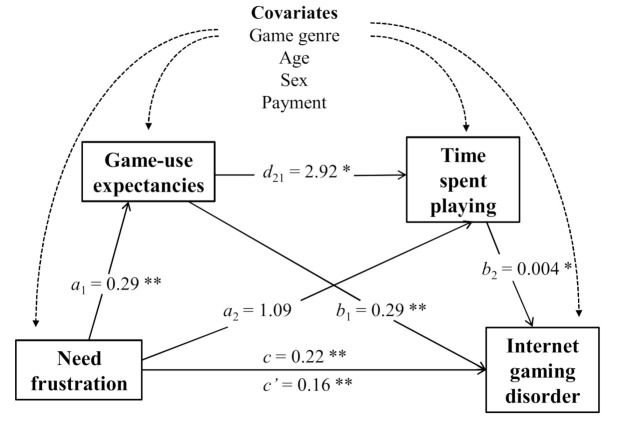
Results of the serial mediation model of the association between need frustration and Internet gaming disorder (* *p* < 0.05, ** *p* < 0.001).

The serial mediation model was significant [F (7, 462) = 33.44, *p* < 0.001] and explained 33.6% of the variance in IGD scores. Specifically, both the total effect of need frustration on IGD (c = 0.106, SE = 0.26, t = 6.17, *p* < 0.001) and the direct effect on game expectancies, the first mediating variable (a1 = 0.295 SE = 0.40, t = 7.29, *p* < 0.001), were significant. The second mediating variable, the direct effect of need frustration on time spent (d21 = 1.09, SE = 0.795, t = 1.37, *p* > 0.05), was not significant. Furthermore, the direct effect of game expectancies (b2 = 0.199, SE = 0.029, t = 6.88, *p* < 0.001) and time spent playing (b3 = 0.004, SE = 0,002, t = 2.68, *p* < 0.05) on IGD scores were also statistically significant. Finally, a significant direct effect of need frustration on IGD was found (c’ = 0.162, SE = 0.26, t = 6.17, *p* = 0.00) when both mediating variables were simultaneously entered into the equation. The indirect effect of need frustration on IGD through game expectancies and time spent playing were significant. These results show that game-use expectancies partially mediate the relationship between need frustration and IGD scores and that game-use expectancies and time spent playing fully mediated the relationship between need frustration and IGD score (see Table 3 and Figure 2).

## 4. Discussion

The present study has applied the construct of the frustration of basic psychological needs in relation to the problematic use of mobile gaming. Previous research had established that playing videogames can be a recreational activity that satisfies the basic psychological needs of autonomy, competence and relatedness [68] but had also suggested that need frustration could have negative consequences in videogamers [30]. However, we are not aware of any published studies that have demonstrated these relationships for gamers who play on their smartphones. Furthermore, we added the mediating effects of a cognitive variable (game expectancies) and a situational variable (time spent playing). We investigated the mediating effects of game expectancies and time spent playing on the relationship between need frustration and IGD; in other words, how personal and environmental antecedents affect the relationship between need frustration and IGD. We hypothesized that need frustration would be directly linked to IGD scores and that it would be mediated by game expectancies and time spent playing. The results confirm most of our hypotheses.

### 4.1. Prevalence of IGD in Mobile Gamers

Considering the significant growth of mobile gaming, it seems important to learn about its potential risks. Following Pontes and Griffiths (2015) [12], we have estimated the prevalence of IGD in our study sample as 3%, which is similar to the 1.9% and 2.6% found in other Spanish samples [66,69]. The results of our study fall within the prevalence rate range reported by other international studies.

### 4.2. The Direct and Mediated Effect of Need Frustration on IGD

The findings reveal a direct effect of need frustration on IGD scores, confirming hypothesis 1. We also observed direct effects of need frustration on game expectancies but not on time spent playing, confirming hypothesis 2 and rejecting hypothesis 3. There is also a direct effect of game expectancies and time spent gaming on IGD, supporting hypotheses 4 and 5. Game expectancies showed a direct effect on time spent gaming, supporting hypothesis 6.

These results suggest that need frustration causes an increase in addiction to mobile gaming and in the expectancy to obtain gratification and avoid boredom but not an increase in the time spent playing. These results show that if mobile gamers’ basic psychological needs are frustrated when they play, because they are forced to act against their volition, cannot be competent or feel rejected or isolated, the consequences are negative—greater probability of experiencing IGD and a greater tendency to seek rewards or evasion when they play. These results are consistent with the suggestion that need frustration can be linked to the development of psychopathology [35] and to a pattern of obsessive gaming [34,36], over which the gamer has little control [46]. Also, previous findings [23] suggest that, despite mobile games being simpler and having limited interfaces, they can produce addictive behaviour, because their users expect them to alleviate feelings of loneliness and to enhance social relatedness. Our results—which indicate a direct effect of need frustration on game expectancies and an indirect effect of need frustration on IGD via expectancies—also support the suggestion of Wegmann and Brand [40] that expectancies play an important role in the addiction process and allow us to extend this conclusion to mobile gaming. They also support the idea that videogames can have a compensatory function, which facilitates IGD, in the same way as has been proposed for online social networks [70].

The findings also reveal a serial mediation effect of game expectancies and time spent playing on the relationship between need frustration and IGD. These results suggest a causal pathway in the IGD scores whereby high need frustration was related to game expectancies, which increased time spent and, thereby, the risk of IGD. This relationship is consistent with the findings of Wegmann and Brand [40], highlighting how this effect is produced—need frustration in gaming leads the player to expect greater gratification, positive expectancies and avoidance expectancies, which in turn lead him or her to play more frequently and for longer. This process results in an addictive use of mobile games. These results confirm the suggestion by Cheng and Leung [4] that mobile gamers play to obtain gratification and avoid boredom, which leads them to spend time gaming in different locations. This result also clarifies the role of time spent gaming, an environmental variable which acts as a mediator [55], in the relationship between need frustration and IGD. These results confirm the importance of time spent playing as a key variable in the problematic use of mobile gaming, as has been proposed for other gaming platforms [53,54,56]. Prolonged time spent gaming has negative psychological and social consequences, because it limits the time available for other activities [58].

Notably, the effect of time spent playing appears to be caused by game expectancies rather than by need frustration, which suggests that participants began spending long periods of time on mobile gaming after they began using cognitive mechanisms to compensate for frustration. In this sense, our results appear to validate the proposal by Wegmann and Brand [40] and suggest that psychological mechanisms are causal antecedents for the effects of other environmental factors and game-related factors. In short, time spent playing is necessary but not sufficient for videogame addiction, as some authors have already suggested [38]. This interpretation is congruent with how time spent is treated in IGD scales [12] and the APA (2013) criteria—it is included as one of several factors that should be considered to evaluate possible addiction.

Our results have been obtained in a sample with a high presence of female videogamers and with micro-payments, in line with the prevalence observed in previous studies [23]. In this sense, the analysis of the effects of the covariates suggest that sex and making micro-payments during the game (males spend more money than females) are two variables to consider in relation to future studies on IGD in mobile video game players. In contrast, game genres and age seem to be unrelated.

Our findings suggest that the theoretical models that have been used to understand problematic Internet use are also applicable to addictive use of smartphone games. It seems likely that IGD—and specifically IGD involving CVG—can be prevented and treated by reducing need frustration and modifying game expectancies. This conclusion should be considered in developing treatment programs for IGD patients.

### 4.3. Limitations

A limitation of our study is its cross-sectional design, which is a constraint on any causal interpretation. Further studies should consider a prospective cohort design to clarify the causal relationships. The use of self-report data from of a self-selected sample (snowball procedure), where reasearchers do not see their subjects is also a limitation that could have introduced biases inherent in any online questionnaire, such as not being able to verify the identity, sex and age (or other sociodemographic data) of the participants.

## 5. Conclusions

In this paper, we set out to investigate, in mobile videogaming, the impact of the frustration of psychological needs on addictive behavior and some mechanisms involved. Our results confirm the negative effects of need frustration on IGD in mobile videogaming. Game-use expectancies mediated the effect of need frustration on IGD, whereas time spent gaming did not; however, it did so indirectly by acting as a mediator for the relationship between game-use expectancies and IGD. The strengths of this study are that our results confirm the important role of cognitions (game-use expectancies) in the development of IGD, previously found in computer videogaming, also in mobile gaming, while controlling important covariates such as game genre, age, sex and payment. These mechanisms (time spent playing) and cognitions (game-use expectancies) are important targets for clinical interventions, even if they are not included in the diagnostic criteria [71]. A weakness of our study is the use of a self-selected sample. Future studies should use sampling procedures by which the identity data provided by the participants can be verified; future studies should also consider the importance of in-game purchases, controlling for age, as well as different game genres.

## Figures and Tables

**Table 1:** Descriptive statistics and Pearson’s correlations matrix for study variables.

	Mean	SD	1	2	3	4	5
1. age	21.73	10.10					
2. payment	14.64	21.39	0.04				
3. need frustration	28.52	9.58	−0.20 **	0.21 **			
4. use expectancies	27.09	9.13	−0.18 **	0.16 **	0.36 **		
5. time spent	75.49	155.74	−0.02	0.14 **	0.13 **	0.21 **	
6. Internet gaming disorder	14.24	6.11	−0.14 **	0.21 **	0.39 **	0.47 **	0.23 **

Note: Note: ** *p* < 0.001.

**Table 2:** Regression coefficients for study variables and covariates over mediators and outcome.

Consequent															
	M1 (Game-Use Expectancies)					M2 (Time Spent Gaming)					Y (Internet Gaming Disorder)				
Antecedent	Coefficient	SE	*p*	LLCI	ULCI	Coefficient	SE	*P*	LLCI	ULCI	Coefficient	SE	*p*	LLCI	ULCI
need frustration	0.295	0.040	0.000	0.216	0.375	1.093	0.795	0.170.	−0.470	2.665	0.162	0.026	0.000	0.111	0.214
use expectancies						2.920	0.864	0.001	1.223	4.617	0.199	0.029	0.000	0.142	0.256
time spent gaming											0.004	0.002	0.008	0.001	0.007
sex	1.784	0.818	0.030	0.176	3.391	11.52	15.29	0.451	−18.52	41.57	1.811	0.505	0.000	0.818	2.80
payment	2.255	0.797	0.005	0.690	3.820	31.01	14.94	0.039	1.639	60.37	1.338	0.496	0.007	0.363	2.31
age	−0.083	0.039	0.031	−0.159	−0.008	0.213	0.072	0.768	−1.20	1.629	−0.021	0.024	0.369	−0.068	0.025
game genre	0.753	0.198	0.000	0.363	1.142	−0.777	3.74	0.836	−8.13	6.58	0.129	1.046	0.296	−0.114	0.373
constant	16.85	1.67	0.000	13.565	20.135	−54.70	34.30	0.113	−121.9	12.99	−6.28	1.13	0.000	−8.52	−4.05
	R^2^ = 0.208 F (5, 464) = 24.44; *p* = 0.000					R^2^ = 0.060 F (6, 463) = 4.93; *p* = 0.000					R^2^ = 0.336 F (7, 462) = 33.44; *p* = 0.000				

**Table 3:** Summary of serial mediation analysis of game expectancies and engagement between need frustration and Internet gaming disorder (IGD) (n = 471, bootstrap = 5000).

Effects			Path								BC a 95% CI	
								B	SE	t	Lower	Upper
Directeffects	need frustration	→	IGD					0.162	0.026	6.17 **		
	need frustration	→	use expectancies					0.295	0.040	7.29 **		
	need frustration	→	time spent					1.09	0.795	1.37		
	use expectancies	→	IGD					0.199	0.029	6.88 **		
	use expectancies	→	time spent					2.92	0.864	3.38 *		
	time spent	→	IGD					0.004	0.002	2.68 *		
	need frustration	→	use expectancies	→	IGD			0.005	0.012		0.037	0.084
Indirecteffects	need frustration	→	time spent	→	IGD			0.005	0.005		−0.002	0.016
	need frustration	→	use expectancies	→	time spent	→	IGD	0.004	0.002		0.001	0.008

Note: * *p* < 0.05, ** *p* < 0.001.
